# The difficult differential diagnosis of BPD look-alikes

**DOI:** 10.1192/j.eurpsy.2021.173

**Published:** 2021-08-13

**Authors:** T. Gondek

**Affiliations:** Early Career Psychiatrists Committee, European Psychiatric Association, Wroclaw, Poland

**Keywords:** Borderline personality disorder, education in psychiatry, differential diagnosis, personality disorders

## Abstract

The traits of Borderline Personality Disorder (BPD) and various other personality or mental disorders may overlap, causing diagnostic difficulties and pitfalls for psychiatrists early in their career. An online survey conducted among psychiatric trainees and young specialists in 2019 showed that only 63% of them think they are well prepared to diagnose BPD. Predispositions such as impulsivity or emotional instability, which commonly are present in BPD, may also be prevalent in such disorders as Antisocial Personality Disorder, Bipolar Disorder, in people misusing psychoactive substances, or in neurodevelopmental disorders such as ADHD. These symptoms can lead to considerable difficulties in global functioning and performing adequate social roles unless appropriate treatment is provided. Therefore, a proper differential diagnosis is crucial in good psychiatric management of people with BPD features. Dr. Gondek will present what BPD symptom domains may be shared with other mental and personality disorders and how to navigate the diagnostic process to set the correct diagnosis in often unobvious clinical presentations of BPD and its look-alikes.

**Disclosure:**

No significant relationships.

